# More than just pronouns – gender-neutral and inclusive language in patient education materials: suggestions for patient education librarians

**DOI:** 10.5195/jmla.2023.1723

**Published:** 2023-07-10

**Authors:** Eleni Philippopoulos

**Affiliations:** 1 eleniphilippopoulos@gmail.com, Information Specialist, Sinai Health, Toronto, Ontario, Canada.

**Keywords:** LGBTQ+, consumer health information services, patient education

## Abstract

Trusted patient education materials are the backbone of an effective consumer health library. However, members of the LGBTQ+ community may not see themselves or their families reflected in many resources due to the gendered and non-inclusive language they are written in. This article outlines some suggestions for concrete actions that patient librarians can take to ensure that their materials are not excluding LGBTQ+ patients.

## INTRODUCTION

Although 4% of Canadians [1] and 7.1% of Americans [2] identify as LGBTQ+, there is an underlying understanding that the health needs of this community are misunderstood [3]. Both in Canada and in the US, LGBTQ+ people face health inequities when it comes to sexual health, substance abuse, mental health, and heart disease [4, 5]. Fear of discrimination prevents LGBTQ+ individuals from accessing healthcare, which can have serious and deadly consequences [6]. Consumer health libraries are meant to be a space where people can take control of their health and improve their health literacy skills. For LGBTQ+ people, doing so may not always be simple, as materials are written using non-inclusive and gendered language and members of this community may not see themselves reflected in the material, further cementing this idea of discrimination. As such, librarians in these spaces should strive to include materials that use gender-neutral and inclusive language in order to make all individuals feel welcome.

## DEFINITION OF TERMS

In the context of this paper, gender-neutral and inclusive language is defined as language that includes everyone, regardless of a person's gender identity, personal identification, and sexual orientation. Gendered and non-inclusive language is language that excludes certain individuals, even though the information being presented is pertinent to and applies to their bodies and their health. Non-inclusive language refers to gender even though a neutral alternative is more appropriate.

## LITERATURE REVIEW

Many libraries, universities, hospitals, and organizations have published guides for healthcare professionals wanting to produce patient education materials. Among the guides reviewed, there are clear instructions on word choice to make the material more understandable and improve overall readability levels [7–15]. These guides discuss the importance of vocabulary, grammar, and paragraph and sentence length. Some guides also note the importance of knowing the audience you are writing for and being sensitive to culture, values and beliefs [12, 16, 17] including the newly-updated Canadian Health Libraries Association Standards for Library and Information Services in Canadian Health & Social Services Institutions [18]. However, the vast majority of these guides fail to mention gender-neutral and inclusive language when advising on the production of patient materials. Instruction on word choice to make the material more inclusive to members of the LGBTQ+ community and gender non-binary individuals is never explicitly stated, with one clear exception. Hamilton Health Services' writing guide makes direct reference to gender and sexual orientation and urges authors to consider the bias presented in the text when these concepts are not taken into account [15]. Other guides make ambiguous references to gender, but do not provide concrete examples or elaborate on important definitions. The Cummings Graduate Institute's web page dedicated to creating patient education materials notes the importance of the material being “appropriate for the audience's age and gender” [16] and the McGill University Health Network and Jewish General Hospital both published guides with sample interview questions for their target audience that asks for information on gender [7, 8]. Instructions from the National Institutes of Health refer to sex but fail to mention gender [12]. This is an important distinction as the two are entirely different concepts.

Articles published in medical journals are more likely to mention gender-neutral language than articles from the field of health and medical librarianship and consumer health information. Research conducted on LGBTQ+ health points to the importance of word choice, both spoken and written, in the clinical setting. To make LGBTQ+ patients feel more welcome, healthcare professionals should make their environments as inclusive as possible by displaying LGBTQ+ symbols and materials [19–29]. The language used in patient education materials presented to patients should be gender-neutral and the content should be written so as to include all patients, regardless of gender and sexual orientation [25, 29–36].

Research suggests that changing the way we address and speak with LGBTQ+ patients in the clinical setting is also important in making them feel included [32, 34, 36]. Emphasis is put on clinicians taking cues from the patients and using the same language and terms they do [37, 38]. LGBTQ+ patients have reported that using non-inclusive language and misgendering is a form of microaggression that can cause them unfortunate stress and harm [22, 24, 39]. It is clear that language, both written and spoken, is a powerful tool within the healthcare context.

## SUGGESTIONS

While librarians have limited control over what takes place in the clinical setting, health librarians involved in patient education and the production of consumer health information can certainly make materials more inclusive and ensure that all patients are represented. Below is a list of suggestions for librarians who advise on creating patient education materials, or those who are involved in the purchasing and organizing of such materials from outside organizations. Suggestions are based on the author's own experiences as a patient librarian in a hospital setting.

## IDENTIFYING LANGUAGE

Identifying health resources that use inclusive and neutral language is a crucial first step in developing patient resources. This author analyzed the brochures on hand in the hospital and library to identify neutral and gendered language instances. In doing this analysis, several patterns emerged:

1. Older/dated brochures and materials tend to use gendered language more than materials written in the last five years. However, there are certainly exceptions.
2. Gendered language goes hand-in-hand with outdated heteronormative views.
3. Gendered language can be avoided altogether by personalizing materials and using the word “you.”
4. Where anatomical terms are discussed, such as making reference to sexual organs, multiple variations of the term are used.

It's important to pinpoint materials that are well-written and understand why they are well-written in order to better set guidelines for the writing of your own materials, or to set clear guidelines for collection development of consumer health collections. It is just as important to identify the materials that you think are not inclusive. Ask yourself what words would make the material more appropriate for everyone. Once you start recognizing the patterns in your own materials, it will be easier to make better choices pertaining to language and spot materials that are inclusive.

## REACHING OUT TO PARTNERS

If you have identified a resource that is well-written, it is worth taking a closer look at it. Is it produced by another hospital or clinic? Is it produced by a community organization or advocacy group? Identifying partners, both within your organization and in the community that you serve, is an important step in becoming inclusive.

In our own organization, we are fortunate to have an LGBTQ+ committee, known as the Pride is Good for Your Health committee, as well as a Diversity and Inclusion department. Contacting similar departments in your own organizations and discussing ideas about language with them is always a good place to start. Consider showing the members of these committees and departments clear examples of non-inclusive language in the organization's materials and examples of inclusive language from other organizations to highlight the differences. If your organization also has a Patient Education committee or department that oversees the production of consumer health materials, reaching out to that committee about updating writing guidelines can also have a great impact on the future standards of the materials produced in-house. If that committee is open to all, joining and being an advocate for inclusive language will also make a difference.

Community resources should also be consulted when discussing language. Oftentimes, there are specialized clinics or organizations that see and treat LGBTQ+ people and have more knowledge about the subject. Start by identifying allied organizations. You can tell a lot about an organization from the information presented on their website, their lists of resources, and the overall mission and purpose statements. For example, an organization that produces a brochure entitled, “What Trans and Nonbinary Patients Can Expect,” is probably an organization that is sensitive to health issues surrounding gender. Compile a list of trusted organizations in your surrounding area and contact them with any questions you might have regarding language. Here are some questions that you may want to ask them:

- Are there certain terms that patients respond to better?
- How do you refer to x or y? Do you have a suggestion for z?
- Have you gotten feedback on your materials? What do patients like or not like about them?
- Are there phrases or terms that should be avoided?

Our library has had contact with a neighboring hospital library that produces excellent inclusive patient education materials. We often point to their materials as an example of how to write inclusive materials. In Ontario, we are fortunate to have Rainbow Health Ontario, an organization whose mission is clearly stated on their website: “Rainbow Health Ontario creates opportunities for the healthcare system to better serve 2SLGBTQ communities.” We have purchased brochures from them, compiled resources based on their reading lists, explored important education opportunities for our staff, and have identified other leaders in the community that can be an asset to us based on their suggestions. Having an organization such as this one to rely on would be a huge asset to any health library looking to spearhead an LGBTQ+ initiative.

## SEEKING OUT FEEDBACK

Most health organizations have active Patient and Family Advisory Councils (PFACs) they rely on to help ensure that patient voices are being heard and act as a body that the organization can stay accountable to. PFACs are made up of diverse populations, who bring with them their unique experiences and backgrounds. The main goal of a PFAC is to teach administrators and clinicians something they may not have known or to encourage them to look at things from a patient's perspective. Engaging with LGBTQ+ members on PFACs can fill gaps in the institution's knowledge about gender-neutral language. Patient librarians should ask to join these PFACs if possible. If not, librarians should ask PFAC facilitators to actively seek out LGBTQ+ feedback on patient materials and comments on the language used.

Currently, our organization has seven active PFACs, including the Fertility LGBTQ2S+ Care Patient Advisory Council and the Women's and Infant's Health Patient and Family Advisory Council. LGBTQ+ people serving on these PFACs have been instrumental in helping change outdated heteronormative and cis-normative language in policies and guidelines at the institutional level. For example, direct conversation with PFAC members resulted in the change of the term VBAC – vaginal birth after cesarean delivery, to the more inclusive TOLAC – trial of labour after cesarean delivery. Likewise, the term breastfeeding has been replaced with infant feeding in an attempt to include all families and parents.

## EDITING FOR INCLUSIVITY

As previously stated, some hospital libraries offer editing services to healthcare professionals writing patient materials [7, 8]. Other organizations have created guides aimed at health writers about the importance of using plain language [12–14, 40]. The Centers for Disease Control & Prevention (CDC) published the Plain Language Thesaurus for Health Communications with the aim of “help[ing] make health information clear and easy to understand”[41]. Our library offers a readability service to healthcare professionals, where the patient librarian works closely with the author to make materials more understandable for audiences. This includes removing medical and technical jargon, reworking sentences, using the active voice, advising on the use of a glossary of terms, and using plain language. We also work with authors to help them understand the goal of the written materials and make sure the takeaway message will be clearly understood by the intended audience.

In the last year, we have also started including inclusivity and neutral language in our editing services. When authors request our editing services, the patient librarian will also scan the text for instances of gendered language using an informal evaluation form (Appendix 1). The form is meant to be a guiding document and offers writers suggestions about neutral terms. In addition, the form allows us to start a conversation with writers about the importance of inclusive materials. This is especially important for areas where sex and gender are often confused and are used interchangeably, such as in gynecology, urology, and lactation (often referred to exclusively in the healthcare environment as breastfeeding).

## DISCLAIMERS

It can be difficult to choose which materials are right for your library when you are trying to be as inclusive as possible. Oftentimes, patient education material that comes from a leading institute and is medically informative and well-written will have instances of gendered language. In our own library, we have and distribute material that is trusted and popular among patients and healthcare practitioners, but that might not be welcoming for all patients because of gendered and heteronormative language. Instead of dismissing these materials altogether, we have chosen to include a disclaimer that makes it clear that we value all patients.

In developing our disclaimer, we started with the hospital's existing statement about health equity [42]. It is a good idea to identify any such statement from your organization or contact the partners that you have already identified in order to write an appropriate statement of your own. After studying other disclaimers and agreeing on the message we wanted to convey, our own disclaimer reads as follows: “This information is intended for EVERYONE. We acknowledge and respect that people use different words to talk about themselves, their families, and their bodies.”

This disclaimer is printed onto labels that are then displayed on the health materials that we found have gendered or heteronormative language. Likewise, for electronic resources, we use a cover sheet that is attached to the information we've retrieved from the internet. This cover sheet also includes our disclaimer about the educational nature and use of the provided material and warns that the information provided should not replace a healthcare professional's counsel. We have a list of trusted LGBTQ+ websites for specific conditions or social issues and do our best to identify online resources that include all individuals. When sending information via email, our electronic signatures also include staff pronouns, indicating that we are a safe space that has taken steps normalize discussions surrounding LGBTQ+ health.

A well-written disclaimer with a clear message shows patients that your organization is an open and understanding space. The goal is to make an effort and show marginalized communities that you care and that you're trying.

## PAY IT FORWARD

If you are committed to being an inclusive organization, your collection development policies should reflect this commitment. If your policies do not explicitly mention marginalized communities, such as the LGBTQ+ community, it is time to rewrite them. If you are a librarian who also oversees a clinical collection or has some say in the purchasing for a clinical collection, all efforts should be made to purchase materials that will help inform health professionals' practice on this subject. It is not enough to simply adjust the language used in materials intended for patients. Healthcare professionals must also change the way they deliver care. The library's job is to purchase resources and maintain a collection that will help them do so.

Our library has purchased a number of books that discuss the issues relating to LGBTQ+ health and that also educate on the language used in the community. Where appropriate, the patient librarian in our library sends healthcare professionals resources from a curated list to help them understand the importance of neutral language in the medical field. The library has created a subject guide exclusively for LGBTQ+ health where healthcare professionals can browse the resources from trusted organizations, including glossaries of LGBTQ+ terms and education materials.

## CONCLUSION

There is no one size fits all approach to creating inclusive materials. However, there needs to be a willingness to adapt and learn. Patient librarians should be aware of issues surrounding non-inclusive language that acts as a deterrent to members of the LGBTQ+ community and act to fix the instances that are within their control. Inclusive language ensures that no one is left out. This is of the utmost importance in a healthcare setting, as healthcare is a right for all.
