# Supplementary material for: More than just pronouns – gender-neutral and inclusive language in patient education materials: suggestions for patient education librarians
Source: J Med Libr Assoc. 2023 Jul 10;111(3):734–9. doi: 10.5195/jmla.2023.1723 (PMC10361550; doi:10.5195/jmla.2023.1723)
Supplement: Supplementary file 1 — Appendix A [file jmla-111-3-734-s01.pdf]

Sinai Health

Appendix A: Evaluation Form – Editing for Inclusivity

This document evaluates your material and makes you think critically about the language you're using. You want to ensure that you're writing in a way that doesn't exclude members of the LGBTQ+ community. If you are unfamiliar with LGBTQ+ terminology and don't know if a term is outdated or offensive, consider familiarizing yourself with the terms using a glossary (https://lgbtqia.ucdavis.edu/educated/glossary). Resources are also available through the library; you can contact a librarian for more information.

Title: ____

Intended audience or targeted patient population (ex: patients with neuropathy, dementia caregivers, spouses of pregnant individuals, etc.):

____

____

Goal of the document (ex: to describe a condition, to instruct on a treatment, to list community resources, etc.):

____

____

____

Do any of the following words appear in the document?

- ☐ Man
- ☐ Woman
- ☐ Girlfriend
- ☐ Boyfriend
- ☐ Wife
- ☐ Husband

Are they essential? Can any of these terms be replaced by **P**atient, **P**erson or **P**artner (**the 3Ps**)?

____

____

Sinai Health

Do any of the following words appear in the document?

- ☐ Motherhood
- ☐ Fatherhood
- ☐ Brother
- ☐ Sister

Are reproductive organs mentioned in your document?

- ☐ Yes
- ☐ No

What terms have you used to name/describe them?

____

____

Do you think the words you used may exclude groups of people (transgender people, non-binary people, intersex individuals, etc.)?

____

Are sexual relationships mentioned in your document?

- ☐ Yes
- ☐ No

What terms have you used to name/describe them?

____

____

Do you think the words you used may exclude groups of people (transgender people, non-binary people, men who have sex with men, women who have sex with women, etc.)?

____

Sinai Health

Would your material be more inclusive if you wrote different documents for different audiences? For example: Breastfeeding Tips and Chestfeeding Tips

____

____

*If you choose to create different materials for different audiences, consider the health-related topics that are important for each group

- ☐ Body / anatomy
- ☐ Medications / hormones
- ☐ Social implications
- ☐ Access to healthcare
- ☐ Other

____

____

____

If you have done everything to make your material more inclusive but don't think you can change anything else without compromising the validity of the information you're presenting, do you think a disclaimer might help?

Example: "This document is intended for all individuals, regardless of sexual orientation and gender. We understand that different people use different terms to describe their identities and their bodies and use terms X, Y and Z for consistency throughout."

____

____

____
